# Regulation of Glycosylphosphatidylinositol-Anchored Protein (GPI-AP) Expression by F-Box/LRR-Repeat (FBXL) Protein in Wheat (*Triticum aestivum* L.)

**DOI:** 10.3390/plants10081606

**Published:** 2021-08-05

**Authors:** Min Jeong Hong, Jin-Baek Kim, Yong Weon Seo, Dae Yeon Kim

**Affiliations:** 1 Advanced Radiation Technology Institute, Korea Atomic Energy Research Institute, 29 Geumgu, Jeongeup 56212, Korea; hongmj@kaeri.re.kr (M.J.H.); jbkim74@kaeri.re.kr (J.-B.K.); 2 Division of Biotechnology, Korea University, 145 Anam-Ro, Seongbuk-Gu, Seoul 02841, Korea; seoag@korea.ac.kr; 3 Institute of Animal Molecular Biotechnology, Korea University, 145 Anam-Ro, Seongbuk-Gu, Seoul 02841, Korea

**Keywords:** *Triticum aestivum*, yeast two-hybrid, F-box protein, SCF complex, TaFBXL, TaGPI-AP

## Abstract

F-box proteins are substrate recognition components of the Skp1-Cullin-F-box (SCF) complex, which performs many important biological functions including the degradation of numerous proteins via the ubiquitin–26S proteasome system. In this study, we isolated the gene encoding the F-box/LRR-repeat (FBXL) protein from wheat (*Triticum aestivum* L.) seedlings and validated that the TaFBXL protein is a component of the SCF complex. Yeast two-hybrid assays revealed that TaFBXL interacts with the wheat glycosylphosphatidylinositol-anchored protein (TaGPI-AP). The green fluorescent protein (GFP) fusion protein of TaFBXL was detected in the nucleus and plasma membrane, whereas that of TaGPI-AP was observed in the cytosol and probably also plasma membrane. yeast two-hybrid and bimolecular fluorescence complementation (BiFC) assays revealed that TaFBXL specifically interacts with TaGPI-AP in the nucleus and plasma membrane, and TaGPI-AP is targeted by TaFBXL for degradation via the 26S proteasome system. In addition, TaFBXL and TaGPI-AP showed antagonistic expression patterns upon treatment with indole-3-acetic acid (IAA), and the level of TaGPI-AP was higher in tobacco leaves treated with both MG132 (proteasome inhibitor) and IAA than in leaves treated with either MG132 or IAA. Taken together, our data suggest that TaFBXL regulates the TaGPI-AP protein level in response to exogenous auxin application.

## 1. Introduction

Ubiquitin is a small globular protein composed of 76 conserved amino acid residues, which attach to the target protein to regulate its degradation by the 26S proteasome. The plant ubiquitin–proteasome system controls the degradation of numerous proteins and plays an important role in plant growth, hormone signaling, abiotic stress response, embryogenesis, and senescence [1]. The process of ubiquitination is mediated by three enzymes: ubiquitin-activating enzyme (E1), ubiquitin-conjugating enzyme (E2), and ubiquitin ligase (E3). To ubiquitinate the target protein, ubiquitin is activated by E1 via the formation of a thioester bond, and the activated ubiquitin is transferred to E2. Then, E3 recruits the activated ubiquitin from E2 and transfers ubiquitin to the target protein for inducing its proteasomal degradation [2]. Over 6% of the genes in the *Arabidopsis thaliana* genome are predicted to encode at least 1400 putative E3 ligases [3,4]. The specificity of target protein degradation is mainly recognized by E3 ligases in a suitable mechanism to respond to environmental alternation, stress response, and regulation of developmental stage during life cycle [5].

CUL1-based E3 ligases or SCF ubiquitin-protein ligases are composed of S-phase kinase-associated protein 1 (Skp1), Cullin 1 (CUL1), F-box protein, and RBX1 [6]. CUL1 acts as a molecular scaffold that brings together RBX1 (which binds to E2 ligase) and Skp1, which serves as an adapter between the F-box proteins and CUL1 links. F-box proteins mediate the ubiquitination and subsequent proteasome-dependent degradation of target proteins in eukaryotic cells [4]. Due to their role in the recognition of the substrate and its recruitment for degradation by the 26S proteasome, F-box proteins show high diversity in plant species. A total of 692, 337, and 779 F-box protein-encoding genes have been identified in *Arabidopsis*, poplar (*Populus* spp.), and rice (*Oryza sativa* L.), respectively, and the plant F-box protein superfamily is divided into 42 families (e.g., FBA, Kelch, leucine-rich repeat (LRR), Tub, WD40, and DUF), which can be distinguished based on the domain organization in the C-terminal region [7]. In plants, F-box protein-mediated ubiquitination and degradation of the target protein are required for the modulation of various developmental and physiological processes, such as seed germination [8], floral organ development [9], abiotic stress response [10,11], plant defense [12,13,14], and phytohormone signal transduction [15,16,17].

The plant hormone auxin is involved in many plant processes and is essential for cell elongation, division, and differentiation, and morphogenesis. The mainstream auxin signal transduction in the nucleus is governed by TIR1/AFB F-box proteins [16]. In the nucleus, auxin binds to TIR1/AFB F-box proteins and promotes the degradation of the Aux/IAA transcriptional repressors through the ubiquitin 26S proteasome system by combinational interactions [18]. Moreover, several signaling mechanisms of auxin are suggested to be regulated in the cell membrane by the plant-specific auxin-binding protein 1 (ABP1), which has been proposed to function as an auxin receptor in the cell membrane [19,20]. ABP1, a 22-kDa glycoprotein, is essential for a wide variety of auxin-regulated processes. A previous study showed that ABP1 plays an important role in clathrin-dependent endocytosis; thus, ABP1 promotes clathrin recruitment for enhancing endocytosis but inhibits the recruitment of clathrin by binding to auxin [21]. Moreover, plasma membrane-localized transmembrane kinases (TMKs) functionally interact with ABP1 in a complex, thus inducing ABP1 to transmit the extracellular auxin signal to the intracellular rho of plants (ROP) signaling component [22]. In addition, since ABP1 lacks a transmembrane domain, it is necessary for another membrane-bound “docking protein” to bind to the plasma membrane [21]. In maize (*Zea mays* L.), glycosylphosphatidylinositol-anchored protein (GPI-AP) has been identified as a candidate docking protein. It is possible that ABP1 contributes to the directional cell growth processes via the plasma membrane-localized GPI-AP, in a similar way as the C-terminal peptide-binding protein 1 (CBP1), and CBP family members interact with the C-terminus of ABP1 to facilitate the secretion of auxin [23,24].

The function of F-box proteins in wheat is largely unknown with respect to wheat developmental stages. Recently, Hong et al. (2020) reported on 1796 F-box genes in the wheat genome classified into various subgroups based on their functional C-terminal domains [25]. Furthermore, transcriptome analysis and expression analysis using reverse transcription-quantitative PCR (RT-qPCR) during various wheat developmental stages revealed that some F-box genes were specifically expressed in the vegetative and/or seed developmental stages [25]. Among them, we select and demonstrate that the wheat (*Triticum aestivum* L.) F-box/LRR-repeat (TaFBXL) protein (TraesCS1B02G275600) interacts with TaGPI-AP both in vivo and in vitro and that TaGPI-AP, which is involved in the response to extracellular auxin to facilitate auxin secretion [23], is degraded by the 26S proteasome. Our results suggest that TaFBXL is a possible regulator of TaGPI-AP in response to exogenous auxin application to facilitate the signaling transduction of the auxin receptor complex.

## 2. Materials and Methods

### 2.1. Plant Materials and Growth Conditions

Wheat (*Triticum aestivum* L.) cultivar Keumkang (IT 213100) developed by the National Institute of Crop Science (RDA, JeonJu-si, Jeonbuk, Korea) was used in this study. Seeds were vernalized and cold stratified at 4 °C for 4 weeks to facilitate germination and were then transferred to an Incu Tissue (72 mm × 72 mm × 22 mm; SPL Life Sciences, Gyeonggi-do, Korea) containing a polypropylene net floating on Hoagland solution (Sigma-Aldrich, Burlington, MA, USA). Then, the seedlings were transferred to pots filled with soil (Sunshine Mix #1; Sun Gro Horticulture, Agawam, MA, USA) and grown in a greenhouse under controlled conditions (25 °C day/20 °C night temperature, 16 h light/8 h dark photoperiod, and 600 µmol m^−2^ s^−1^ light intensity). Plant samples were collected at different developmental stages, determined according to the Zadoks growth scale [26]: leaf at Z13 (three leaves emerged; Stage 1), leaf at Z24 (main stem and four tillers; Stage 2), leaf at Z51 (leaf at the tip of ear just visible, booting stage; Stage 3), spikelets at Z61 (beginning of anthesis; Stage 4), spikelets at Z73 (early milk development; Stage 5), spikelets at Z83 (early dough; Stage 6), and spikelets at Z91 (hard grain; Stage 7). To determine the effect of abiotic stress on *TaFBXL* expression at the fully expanded 3rd leaf stage (Zadok scale 13), seedlings were sprayed with 100 μM of indole-3-acetic acid (IAA) mixed with 0.2% Tween 20 (1 mL per plant) [27,28]. Then, seedlings were collected at 0, 2, 6, and 12 h after the spray treatment and stored at −80 °C until needed for RNA extraction.

### 2.2. Gene Cloning and Gene Expression Analysis

Total RNA was extracted from each sample using TRIzol reagent (Invitrogen, Waltham, MA, USA) and was treated with DNase I (New England Biolabs, MA, USA) to remove any traces of contaminating DNA. First-strand cDNA was synthesized from 1 μg of total RNA using a Power cDNA Synthesis Kit (iNtRON Biotechnology, Gyeonggi-do, Korea). Full-length coding sequences (CDSs) of *TaFBXL* (TraesCS1B02G275600), *TaGPI-AP* (TraesCS2D02G145400), and *TaABP1* (TraesCS5A02G098900) genes were amplified using gene-specific primer sets designed on the basis of the wheat reference sequence (version 45) available at Ensembl Plant (https://plants.ensembl.org/index.html, accessed 20 March 2020) (Table S1). PCR for gene isolation was performed under the following thermocycling conditions: initial denaturation at 94 °C for 10 min, followed by 31 cycles of denaturation at 94 °C for 1 min, annealing at a gene-specific temperature for 2 min, and elongation at 72 °C for 1 min, and a final extension at 72 °C for 5 min. PCR products were cloned into the pCR8/GW/TOPO cloning vector (Thermo Fisher Scientific, Waltham, MA, USA) and sequenced. The amino acid sequence of TaFBXL was analyzed using InterProScan (https://www.ebi.ac.uk/interpro/search/sequence-search, accessed 15 April 2020).

To analyze gene expression analysis, RT-qPCR was performed in a 48-well plate (Eco Real-Time PCR System, Illumina, San Diego, CA, USA) using 1 μL of reverse-transcribed cDNA and 2 × TB Green Premix Ex Taq II (Tli RNaseH plus; Takara, Kusatsu, Shiga, Japan). The RT-qPCR cycling conditions were as follows: initial denaturation at 95 °C for 5 min, followed by 40 cycles of denaturation at 95 °C for 10 s and annealing and extension at 65 °C for 30 s. Three biological replicates of cDNA from every single plant and spikelet in Stage 1–4 and Stage 5–7 stage, respectively, were used for RT-qPCR experiments. Additionally, the *Actin* (AB181991) gene was used as an endogenous control for relative quantification (RQ) of *TaFBXL* expression. RQ is a fold change in each sample, compared to the calibrator (Stage 1), which has an RQ value of 1. Standard curves were prepared for both the target and endogenous reference. For each experimental sample, the amount of target and endogenous reference was determined from the appropriate standard curve. Then, the target amount was divided by the endogenous reference amount to obtain a normalized target value. Each normalized target value was divided by that of the calibrator to generate the relative expression levels. The primer sets for RT-qPCR are listed in Table S1.

### 2.3. Yeast Two-Hybrid (Y2H) Assay and Library Screening

A wheat (*T. aestivum* L.) cDNA library was constructed for Y2H screening. Total RNA was extracted from leaves at Z13 (Stage 1) using the TRIzol reagent. Then, a wheat cDNA library was constructed from 2 μg total RNA using the Make Your Own Mate & Plate™ Library System (Clontech, Takara, Kusatsu, Shiga, Japan), according to the manufacturer’s protocol. All colonies were pooled with the freezing medium (YPD agar medium with 25% glycerol), and 1 mL aliquots of the cDNA library were stored at −80 °C. To perform the Y2H screen, the full-length coding sequence (CDS) of *TaFBXL* was cloned into the pGBKT7-GW vector (bait) using the Gateway LR recombination reaction (Thermo Fisher Scientific, Waltham, MA, USA). The pGBKT7-*TaFBXL* was transformed into yeast (*Saccharomyces cerevisiae*) strain Y2HGold using the Matchmaker Gold Y2H System (Clontech, Takara, Kusatsu, Shiga, Japan), according to the manufacturer’s protocol. Y2HGold cells harboring bait vector were maintained in minimal synthetic defined (SD) medium lacking tryptophan (SD/-Trp). Then, bait strain and cDNA library (prey) were mixed and incubated at 30 °C with shaking at 30–50 rpm for 20–24 h. The mating cultures were spread onto plates containing double dropout (DDO) SD medium lacking leucine (Leu) and Trp (SD/-Leu/-Trp) but containing X-alpha-galactosidase (X-α-Gal) and Aureobasidin A (AbA) (DDO/X/A). Colonies appeared after approximately 5–7 days and were transferred to quadruple dropout medium (SD/-adenine [Ade]/-histidine [His]/-Leu/-Trp) supplemented with X-α-Gal and AbA (QDO/X/A). Colonies that grew on QDO/X/A plates were considered as candidates representing positive interactions between bait and prey proteins. Yeast colony PCR using Matchmaker Insert Check PCR Mix 2 (Clontech, Takara, Kusatsu, Shiga, Japan) was performed to select positive clones. The selected prey plasmids were rescued using the Easy Yeast Plasmid Isolation Kit (Clontech, Takara, Kusatsu, Shiga, Japan). The prey inserts were sequenced and annotated using the National Center for Biotechnology Information GenBank database.

### 2.4. Co-Transformation and β-Galactosidase (β-Gal) Activity Assay

CDSs of *TaFBXL*, *TaGPI-AP*, and *TaABP1* were cloned into the pGBKT7 vector using the Gateway system [29]. Using the lithium acetate method, each of the *TaFBXL*, *TaGPI-AP*, and *TaABP1* constructs was co-transformed into the yeast strain AH109, along with one of six TaSKP (*TaSKP1*–*TaSKP6*) CDSs previously cloned into the pCR8/GW/TOPO vector (Thermo Fisher Scientific, MA, USA) [30]. Transformants were plated onto SD/-Leu/-Trp or SD/-Ade/-His/-Leu/-Trp, supplemented with X-α-Gal to detect prey–bait interactions. AH109 cells co-transformed with SV40 large T antigen (pGADT7-T) and p53 (pGBKT7-53) and AH109 cells co-transformed with SV40 large T antigen (pGADT7-T) and Lamin-C (pGADT7-Lam) were used as positive and negative controls, respectively.

To validate the strength of a protein–protein interactions, quantitative β-Gal activity was assayed using 2-nitrophenyl β-D-galactopyranoside (ONPG; Sigma) as a substrate, according to the Clontech yeast protocols handbook (protocol no. PT3024-1). Yeast cells were grown overnight at 30 °C in SD selective medium. Then, 2 mL of the cell culture was added to 8 mL of yeast extract peptone dextrose (YPD) media, and the culture was grown until the optical density of the culture at 600 nm (OD_600_) reached 0.8. Thereafter, yeast cells were centrifuged and re-suspended in Z-buffer (60 mM Na_2_HPO_4_, 40 mM NaH_2_PO_4_, 10 mM KCl, and 1 mM MgSO_4_ [pH 7.0]). The yeast cell pellets were lysed via freeze–thaw cycles. All reactions were initiated by the addition of Z-buffer (containing β-mercaptoethanol and 4 mg/mL ONPG) to the samples. The reactions were incubated at 30 °C for 30 min and then stopped by adding chilled 1 M Na_2_CO_3_ for the development of yellow color. Absorbance was measured at 420 nm [31], and *β-Gal activity* was calculated according to the following formula:

$$\beta -Galactivity=\frac{A_{420}}{\left(T\times V\times A_{600}\right)}\times 1000$$

where *T* is the time of reaction (min) and *V* is the volume of culture used in the assay (mL).

### 2.5. Subcellular Localization Analysis and Bimolecular Fluorescence Complementation (BiFC) Assay

To conduct subcellular localization analysis, the open reading frames (ORFs) of *TaFBXL*, *TaGPI-AP*, and *TaABP1* were cloned into a pMDC43 vector [29] using LR clonase (Thermo Fisher Scientific, Waltham, MA, USA) to generate N-terminal fusions with the enhanced *green fluorescent protein* (*GFP*) gene. The resulting plasmids, *35S:GFP-TaFBXL*, *35S:GFP-TaGPI-AP*, and *35S:GFP-TaABP1* were introduced into *Agrobacterium tumefaciens* strain GV3101 using the freeze–thaw method. The transformed GV3101 cells harboring *35S:GFP-TaFBXL* and *35S:GFP-TaGPI-AP* were grown in Luria–Bertani medium until the OD_600_ of the culture reached 1.0. The cells were harvested by centrifugation at 5000× *g* for 10 min at room temperature, and the cell pellets were resuspended in infiltration buffer (10 mM MES, 10 mM MgCl_2_, and 200 mM acetosyringone [pH 5.6]). The *Agrobacterium* cell suspension was infiltrated into tobacco (*Nicotiana benthamiana*) leaves using a syringe.

To conduct the BiFC assay, the ORFs of *TaFBXL*, *TaGPI-AP*, and *TaABP1* were cloned into the destination vectors pGTQL1211-YN (GFP-N) or pGTQL1221-YC (GFP-C) using the Gateway system [29]. Then, all constructs were introduced into *A. tumefaciens* strain GV3101 and infiltrated into tobacco leaves, as previously described [32]. Fluorescence in tobacco leaves was detected at 72 h post-infiltration (hpi) using a confocal laser-scanning microscope (Zeiss LSM800, Oberkochen, Germany).

### 2.6. MG132 Treatment and Western Blot Analysis

*A. tumefaciens* GV3101 carrying the *35S:GFP-TaGPI-AP* construct was infiltrated into the leaves of 4-week-old tobacco (*N*. *benthamiana*) plants and incubated at 22 °C for 48 h. To perform proteasome inhibitor treatments, leaves of independent tobacco plants were injected with 50 μM MG132 and sprayed with 100 μM IAA. Tobacco (*N*. *benthamiana*) leaves were collected at 0, 3, 6, and 12 hpi, and total protein was extracted from the leaves using the protein extraction buffer (Elpis Biotech, Daejeon, Korea). The protein concentration was determined using the Bradford Assay Kit (Takara, Shiga, Japan). To perform Western blot, the protein concentrations of all samples were adjusted to 0.5 µg/µL, following which 5 µg of total extracted proteins from tobacco leaves were separated on 12% acrylamide gel using sodium dodecyl sulfate–polyacrylamide gel electrophoresis (SDS-PAGE) and then transferred onto nitrocellulose membranes using the iBlot 2 Dry Blotting System (Thermo Fisher Scientific, Waltham, MA, USA). Membranes were incubated with rabbit anti-GFP antibody (primary antibody; Abcam, Cambridge, UK). Then, HRP-conjugated goat anti-rabbit antibody (secondary antibody; Thermo Fisher Scientific, Waltham, MA, USA) was used as a secondary antibody to generate the light signal. The membrane was visualized using Amersham ECL Western Blotting Detection Reagent (GE Healthcare, Chicago, IL, USA), and chemiluminescence was recorded using the iBright CL1000 Imaging System (Invitrogen, Waltham, MA, USA).

## 3. Results

### 3.1. TaFBXL Isolation and RNA Accumulation Patterns

**Figure 1:**
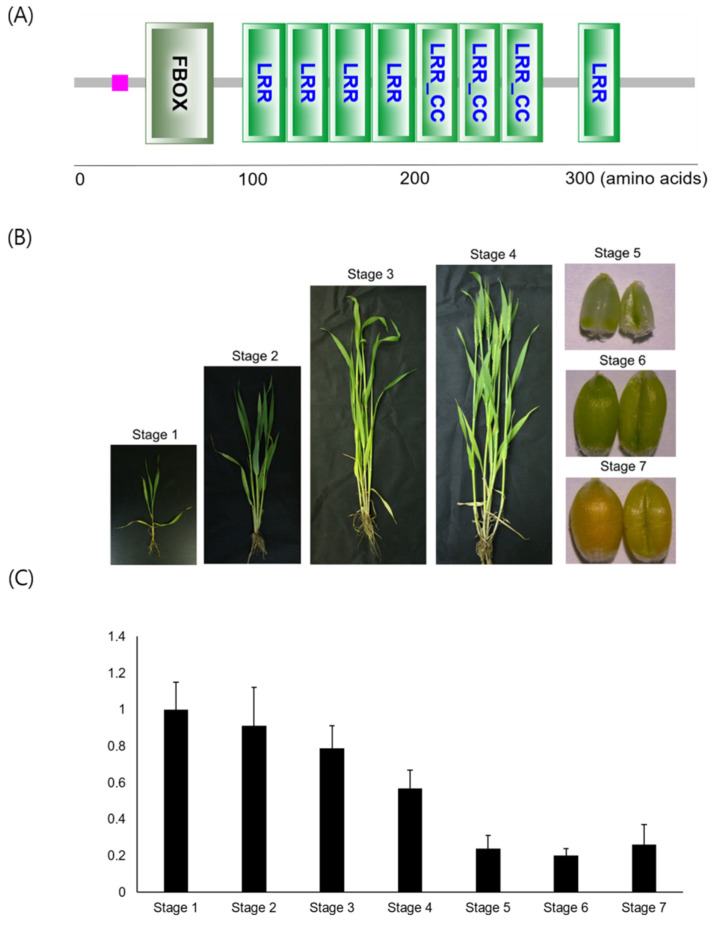
Analysis of the TaFBXL protein structure and *TaFBXL* gene expression: (**A**) domain structure of TaFBXL. Domains identified by the hidden Markov model algorithms of SMART or PFam include F-box motif, leucine-rich repeats (LRR), and leucine-rich repeat and cysteine-containing (LRR_CC) motif; (**B**) images showing different developmental stages of wheat used for reverse transcription-quantitative polymerase chain reaction (RT-qPCR). Stage 1, leaf at Z13 (three emerged leaves); Stage 2, leaf at Z24 (main stem and four tillers); Stage 3, leaf at Z51 (leaf at tip of ear just visible, booting stage); Stage 4, spikelets at Z61 (beginning of anthesis); Stage 5, spikelets at Z73 (early milk development); Stage 6, spikelets at Z83 (early dough); Stage 7, spikelets at Z91 (hard grain); (**C**) transcript levels of *TaFBXL* at seven different developmental stages of wheat, determined by RT-qPCR. RT-qPCR was performed with three biological replicates, and each bar represents mean ± SD for average *n* = 3 independent experiments.

The *TaFBXL* gene (TraesCS1B02G275600) was isolated from wheat seedling cDNA, based on the wheat reference genome sequence provided by the International Wheat Genome Sequencing Consortium. The *TaFBXL* ORF was 1062 bp in size and was predicted to encode a 379-aa protein (Table S2). The amino acid sequence of TaFBXL was analyzed by the Simple Modular Architecture Research Tool (SMART) to identify and annotate the protein domains and to analyze the protein domain architecture [33]. TaFBXL consisted of one F-box domain at the N terminus (SMART accession: SM000256), five Leu-rich repeat domains (SMART accession: SM000370), and three Leu-rich repeat, cysteine (Cys)-containing domains (SMART accession: SM000367) (Figure 1A and Table S3). Then, the expression of *TaFBXL* was analyzed by RT-qPCR in wheat seedlings at different developmental stages including leaf at Z13 (three leaves emerged; Stage 1), leaf at Z24 (main stem and four tillers; Stage 2), leaf at Z51 (leaf at the tip of ear just visible, booting stage; Stage 3), spikelets at Z61 (beginning of anthesis; Stage 4), spikelets at Z73 (early milk development; Stage 5), spikelets at Z83 (early dough; Stage 6), and spikelets at Z91 (hard grain; Stage 7) (Figure 1B). *TaFBXL* showed high expression during vegetative growth (Figure 1C).

### 3.2. TaFBXL Is a Component of the SCF Complex

**Figure 2:**
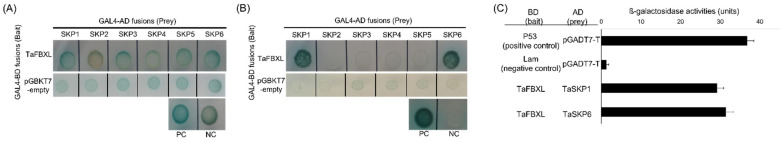
Analysis of interactions between TaFBXL and TaSKP by yeast two-hybrid assays: (**A**,**B**) co-transformants grown on SD/-Leu/-Trp (**A**) or SD/-Ade/-His/-Leu/-Trp (**B**) medium supplemented with X-α-Gal. SV40 large T antigen (pGADT7-T)/p53 (pGBKT7-53) and SV40 large T antigen (pGADT7-T)/Lamin-C (pGBKT7-Lam) were used as positive (PC) and negative controls (NC), respectively. The pGBKT7-empty vector was also used as negative control; (**C**) strength of TaFBXL–TaSKP interactions measured by performing β-Gal assays using 2-nitrophenyl β-D-galactopyranoside as a substrate. Each bar represents mean ± SD for average *n* = 3 independent experiments.

The SCF ubiquitin-protein ligase is an E3 ligase, and the F-box protein is linked to the core complex through its interaction with Skp1, which is important for protein ubiquitination [34]. Previous reports demonstrated that wheat F-box proteins interact with SKP1-like protein to form an SCF complex [35,36]. To confirm that TaFBXL protein is a component of the SCF complex, we tested direct interaction between TaFBXL and TaSKPs by performing Y2H assays. The TaFBXL gene was fused with the GAL4 DNA-binding domain in the pGBKT7 vector (bait), and six TaSKP sequences were fused to the activation domain of GAL4 in the pGADT7 vector. All interactions between TaFBXL and TaSKPs were tested on the DDO/X/A medium after co-transformation (Figure 2A), whereas only two combinations of TaFBXL and TaSKPs were grown on the QDO/X/A medium (Figure 2B). TaFBXL showed interaction with TaSKP1 and TaSKP6. Then, the β-Gal assay was performed to quantify the relative intensity of the protein–protein interactions. Consistent with the results of the Y2H assay, the β-Gal activities of TaFBXL–TaSKP1 and TaFBXL–TaSKP6 interactions were 28.4 and 34.0 units, respectively (Figure 2C).

### 3.3. TaFBXL Interacts with TaGPI-AP

**Figure 3:**
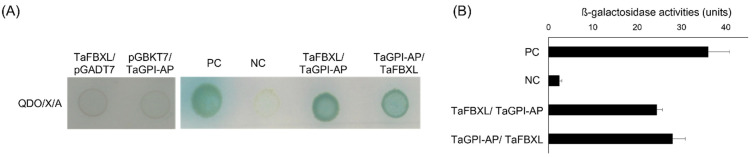
Interaction between TaFBXL and TaGPI-AP determined by yeast two-hybrid (Y2H) assays: (**A**) results of the Y2H assay showing the interaction between TaFBXL and TaGPI-AP; (**B**) quantification of the strength of interaction between TaFBXL and TaGPI-AP by performing the β-Gal assay. The experiments were performed in triplicates. Error bars indicate ± SD of three independent experiments. PC: positive control (pGBKT7-p53/pGADT7-T), NC: negative control (pGBKT7-Lam/pGADT7-T). The pGADT7-empty or pGBKT7-empty vector was also used as negative controls (left panel). Each bar represents mean ± SD for average *n* = 3 independent experiments.

Y2H assays were performed using the wheat seedling cDNA library to identify proteins that interact with TaFBXL. Approximately, 3.87 × 10^7^ transformants were tested, and 50 positive clones were identified in two consecutive rounds of testing (first round: DDO, second round: QDO + X-α-Gal). These were amplified by colony PCR and sequenced. Among them, only six proteins were characterized after exclusion of the false positives and out-of-frame clones. Six clones were selected based on BLASTX alignment of their deduced amino acid sequences (Table 1). For further analysis, co-transformation of TaFBXL and full-length TaFBXL-interacting proteins was performed. Among the putative binding partners of TaFBXL, GPI-AP was found to be a highly reliable positive candidate. This study mainly focuses on GPI-AP, which plays key roles in a wide variety of biological processes occurring at the interface of the plasma membrane and cell wall, such as signaling, cell wall metabolism, cell wall polymer cross-linking, and plasmodesmatal transport [23,37]. To validate the interaction between TaFBXL and TaGPI-AP, yeast co-transformation assay was conducted with the combinations of pGBKT7:TaFBXL and pGADT7:TaGPI-AP, and its reciprocal interaction was assessed by the combination of pGBKT7:TaGPI-AP and pGADT7:TaFBXL. The results of co-transformation assays confirmed the interaction between TaFBXL and TaGPI-AP, as yeast colonies showed growth on QDO/X/A media in both combinations (Figure 3A). Consistent with these results, strong β-Gal activity was detected in both co-transformation assays (Figure 3B).

### 3.4. Subcellular Localization and Interaction between TaFBXL and TaGPI-AP

**Figure 4:**
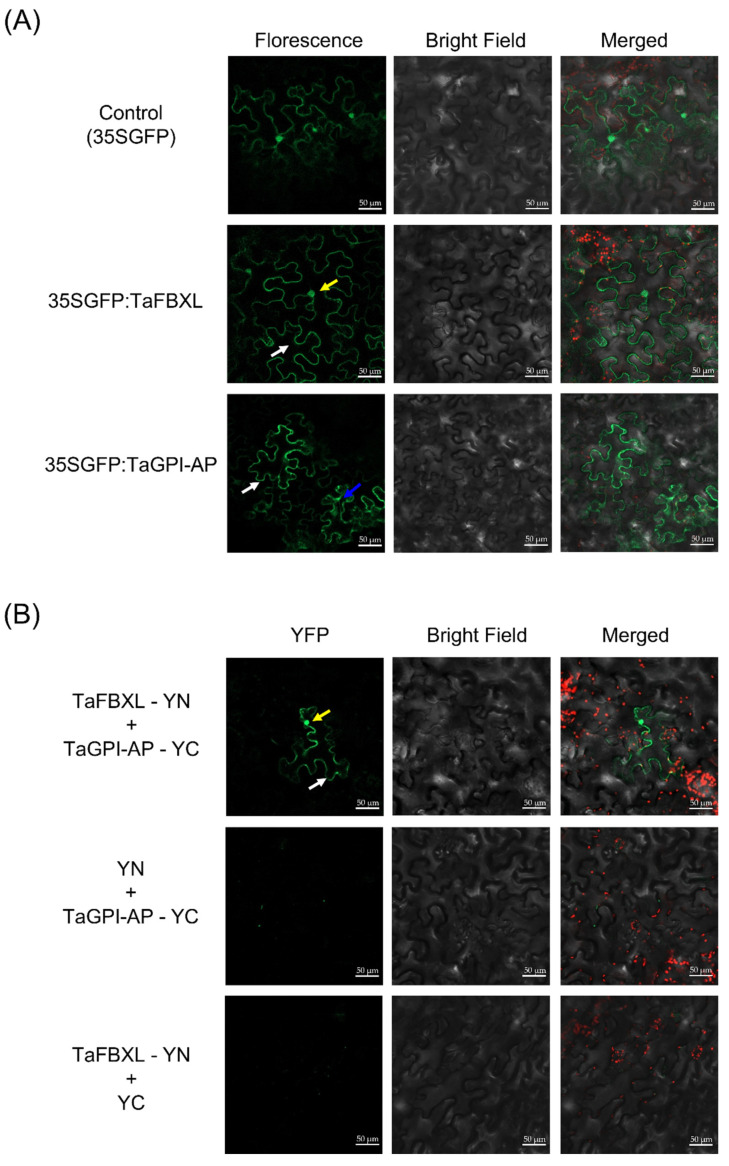
Analysis of the subcellular localization of GFP:TaFBXL and GFP:TaGPI-AP fusion proteins and the interaction between these proteins: (**A**) subcellular localization of GFP:TaFBXL and GFP:TaGPI-AP fusion proteins visualized by confocal microscopy. Tobacco (*N*. *benthamiana*) leaf epidermal cells were infiltrated with *A. tumefaciens* carrying the GFP:TaFBXL fusion construct driven by the 35S promoter. Yellow arrow: nucleus, white arrows: plasma membrane, blue arrow: cytosol; (**B**) results of the bimolecular fluorescence complementation assay used to detect the interaction between TaFBXL and TaGPI-AP in agroinfiltrated tobacco (*N*. *benthamiana*) leaves. Yellow arrow: nucleus, white arrow: plasma membrane. YN, N-terminal region of GFP; YC, C-terminal region of GFP.

To explore the biological function of TaFBXL and TaGPI-AP, we investigated the subcellular localization of these proteins. Vectors carrying GFP:TaFBXL and GFP:TaGPI-AP GFP:TaABP1 fusions were constructed and then agroinfiltrated into tobacco (*N*. *benthamiana*) leaves. The fluorescence signal of GFP:TaFBXL was detected in the nucleus and plasma membrane (Figure 4A), whereas that of GFP:TaGPI-AP was observed in the cytosol and probably also the plasma membrane. To examine the interaction between TaFBXL and TaGPI-AP in plants, we performed BiFC assays. TaFBXL was fused to the N-terminal fragment of GFP (EGFP-N) to generate the pGTQL1211-TaFBXL construct, while TaGPI-AP was fused to the C-terminal fragment of GFP (EGFP-C) to generate the pGTQL1221-TaGPI-AP construct. The BiFC assay revealed an interaction between TaFBXL and TaGPI-AP in the nucleus and plasma membrane (Figure 4B).

### 3.5. TaGPI-AP Interacts with TaABP1

**Figure 5:**
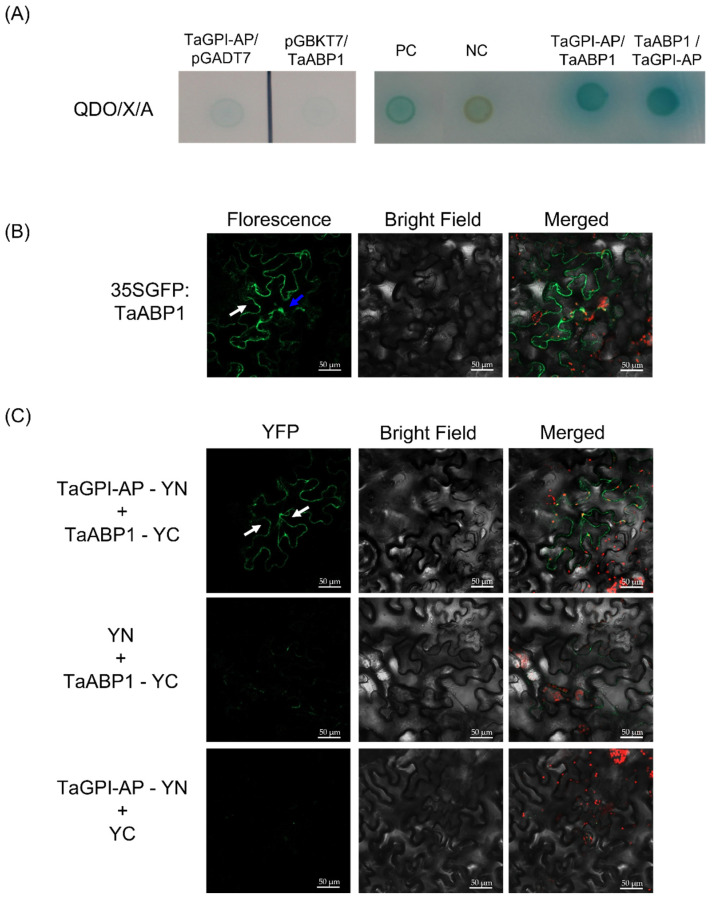
Analysis of the interaction between TaGPI-AP and TaABP1 by yeast two-hybrid (Y2H) and bimolecular fluorescence complementation (BiFC) assays: (**A**) results of the Y2H assay showing the interaction between TaGPI-AP and TaABP1. PC: positive control (pGBKT7-p53/pGADT7-T), NC: negative control (pGBKT7-Lam/pGADT7-T). The pGADT7-empty or pGBKT7-empty vector was also used as negative controls (left panel); (**B**) subcellular localization of 35SGFP:TaABP1. Confocal images of tobacco (*N*. *benthamiana*) leaf epidermal cells expressing the GFP fusion of TaABP1 are shown. White arrow: plasma membrane, blue arrow: cytosol; (**C**) interaction between TaGPI-AP and TaABP1 proteins in the BiFC assay. TaGPI-AP fused to the N-terminal YFP, and TaABP1 fused into the C-terminal YFP. White arrows: plasma membrane.

A previous study indicated that a GPI-AP protein named CBP1 interacts with ABP1 in maize [24]. To demonstrate the role of TaGPI-AP as a membrane-bound docking protein combined with ABP1, we confirmed the interaction between TaGPI-AP and TaABP1 proteins using Y2H and BiFC assays. The interaction between TaGPI-AP and TaABP1 was confirmed by yeast co-transformation analysis (Figure 5A). TaABP1 localized to the cytosol and probably also plasma membrane (Figure 5B), and weak interaction was detected between TaGPI-AP and TaABP1 in the plasma membrane via the BiFC assay (Figure 5C).

### 3.6. Degradation of TaGPI-AP in 26S Proteasome

**Figure 6:**
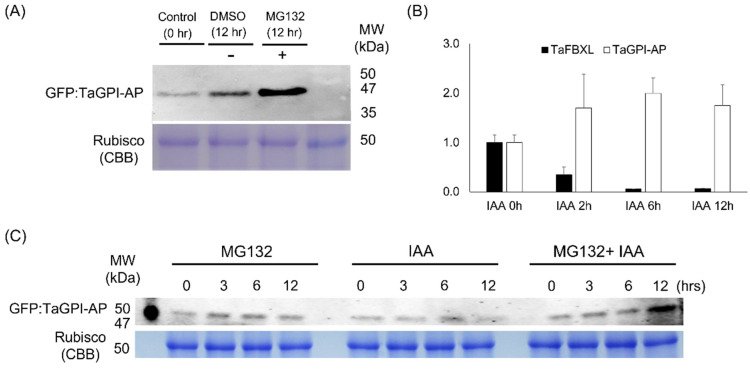
Inhibition of protein degradation by 26S proteasome in plants: (**A**) Western blot analysis using total protein extracted from transiently transformed tobacco (*N*. *benthamiana*) leaves expressing GFP:TaGPI-AP and treated with (+) or without (-) 50 µM MG132. The GFP:TaGPI-AP protein was detected using an anti-GFP antibody. Coomassie Brilliant Blue (CBB) staining was used to verify the amount of protein loaded; (**B**) RNA accumulation patterns of *TaFBXL* and *TaGPI-AP* under indole-3-acetic acid (IAA) treatment using RT-qPCR. Seedlings with fully expanded 3rd leaf were collected at 0, 2, 6, and 12 h after treatment with 100 μmol IAA. RT-qPCR was performed with three biological replicates. Each bar represents the mean ± SD of three independent experiments (*n* = 3); (**C**) Western blot analysis of protein extracts prepared from transiently transformed tobacco (*N*. *benthamiana*) leaves expressing GFP:TaGPI-AP treated with 50 μM MG132. CBB indicates the CBB staining of Rubisco’s large subunit as a loading control in 5 µg of total protein.

To determine whether TaGPI-AP is degraded by the 26S proteasome in vivo, total proteins were extracted from tobacco (*N*. *benthamiana*) leaves infiltrated with *A. tumefaciens* in the presence or absence of MG132 (proteasome inhibitor). Western blotting with an anti-GFP antibody was used to validate the production of the foreign protein. The level of TaGPI-AP in leaves treated with MG132 was higher than that in leaves not treated with MG132 at the same point (Figure 6A). Previous studies showed that GPI-AP interacts with the C-terminus of ABP1 to facilitate the secretion of auxin by providing a docking position on the cell membrane to ABP1 [38]. Therefore, we evaluated the effect of IAA treatment on the RNA accumulation patterns of *TaFBXL* and *TaGPI-AP* and on the levels of the encoded proteins. *TaFBXL* and *TaGPI-AP* showed opposite RNA accumulation patterns under the IAA treatment; when *TaFBXL* was downregulated, *TaGPI-AP* was upregulated and vice versa (Figure 6B). Consistent with the results shown in Figure 6A, the level of TaGPI-AP was enhanced by treatment with the 26S proteasome inhibitor, MG132. In the absence of MG132, the accumulation of TaGPI-AP was reduced compared with DMSO-treated samples at the same time point. A higher accumulation of TaGPI-AP was found in samples treated with both, MG132- and IAA, than in samples treated with either MG132 or IAA (Figure 6C).

## 4. Discussion

Many members of the *F-box* gene family are induced by intracellular signals, environmental changes, and abiotic and biotic stress conditions [39]. The diverse expression patterns of a large number of *F-box* genes suggest that these genes play diverse roles in plant growth and development and in the response to cellular and extracellular cues [40]. Most F-box proteins studied to date participate in the recognition of target proteins for ubiquitination and 26S proteasome-mediated protein degradation as a component of the SCF complex [41]. FBX proteins contain an F-box motif at the N terminus and diverse C-terminal domains, such as LRR, Kelch, WD-40, Armadillo (Arm), tetratricopeptide repeats (TPRs), Tub, actin, DEAD-like helicase, and jumonji (JmjC), which recognize the target protein [42]. FBXL is a large subfamily of the plant FBX family, and 160, 61, and 46 FBXLs have been identified in *Arabidopsis*, rice, and maize, respectively [14,39,42]. Previous studies showed that plant FBXLs mediate target protein degradation in response to developmental and hormonal signals [43,44]. In addition, LRR domains identified at the C-terminus of TaFBXL constitute a domain superfamily found in animals, yeast, protists, bacteria, and plants. The LRR domain is characterized by the presence of tandem repeats of 18–29-aa long Leu-rich motif [45]. Although LRRs are found in diverse proteins, their function is highly conserved across plant species; LRRs provide a platform for specific protein–protein interactions [46] and are critical for protein function, as they are involved in either ligand recognition or docking to other components of the pathway [47]. However, the function of FBXL in wheat remains largely unknown.

To investigate the function of FBXL in wheat, we isolated the CDS of the *TaFBXL* gene from the cDNA of wheat seedlings. Sequence analysis revealed that TaFBXL contains a conserved F-box domain at the N terminus and eight LRRs at the C terminus, which are potentially involved in the recognition of the target protein (Figure 1A and Table 1). We confirmed the interaction between TaFBXL and TaSKPs, thus validating that the N terminus of the F-box domain interacts with TaSKPs, resulting in the formation of the SCF complex for 26S proteasome-mediated ubiquitination of the target protein. TaFBXL showed strong interaction with TaSKP1 and TaSKP6 (Figure 2), indicating that TaFBXL possessing F-box domain interacted with TaSKPs as a component of the SCF complex. The previous reports identified the putative F-box genes and six TaSKPs in wheat and showed protein–protein interactions between F-box proteins and TaSKPs to verify whether they are the components of wheat SCF complexes [30,35,36]. Interestingly, F-box proteins did not interact with all TaSKPs and showed different interaction patterns. Although all deduced TaSKPs contained an SKP1 component and POZ domain (IPR016073, InterProScan), TaSKP1 and TaSKP6 showed the greatest similarity with 97% identical residues, while TaSKP2 showed lower conservation at the amino acid sequence level (less than 35% identical residues), compared with other TaSKPs [36]. The variance of sequence composition may influence differently on both interactions of TaSKPs with F-box protein and protein functions.

IAA plays a crucial role in the regulation of a wide range of plant growth and developmental processes [48]. In the last few decades, ABPs have been identified and proposed to play a key role in auxin perception at the plasma membrane [19,49]. ABP1 localizes to the plasma membrane, where it mediates rapid electrophysiological and cell physiological responses to auxin [50] and acts as a modulator of clathrin-mediated endocytosis and microtubule orientation through its action on the ROP family of GTPases [51,52]. GPI-APs mediates the posttranslational glycolipid modification of numerous cell surface proteins in eukaryotes [53]. GPI biosynthesis starts with a lipid molecule at the rough side of the ER triggered by a hydrophobic signal sequence of GPI-AP at the C terminus. GPI-AP is further modified in the ER and Golgi apparatus, that hydrophobic signal at the C terminus is cleaved at the ω position and replaced by various types of glycans and lipids for remodeling. After various maturation steps, GPI-AP is transported to the cell membrane [53,54,55]. Interestingly, maize GPI-AP, named CBP1, directly interacts with extracellular auxin by binding to ABP1, and the CBP1–ABP1 complex is recognized by the cell surface reporter, TMM1, leading to a quick auxin response [23,56,57]. Consistent with previous reports, we showed that TaGPI-AP localizes to the plasma membrane and that TaFBXL localizes to the nucleus and plasma membrane. Moreover, our BiFC and yeast co-transformation assays showed that TaGPI-AP interacts with TaABP1 at the plasma membrane.

However, the role of ABP1 in auxin signaling has remained controversial, and its mode of action remains unclear. The *abp1* null alleles generated using the CRISPR-Cas technology showed indistinguishable phenotypes during flowering and did not show a strong or rapid response to auxin in the plasma membrane of wild-type plants [58]. In addition, studies showed that membrane depolarization and endocytosis inhibition are ABP1-independent responses, and auxin-induced plasma membrane depolarization is dependent on the auxin influx carrier AUX1 [59]. On the other hand, the *Arabidopsis* GPI-AP protein participates in various biological processes, such as signal transduction by associating with cell surface receptors and/or extracellular ligands, developmental processes, and stress and immune responses [39]. *Arabidopsis* GPI-AP (*SKU5*) with auxin-related extracellular protein-ligand ABP1 could be recognized by the cell surface receptor kinase TMK1 and activate intracellular signaling components [60]. Furthermore, GPI-AP (*SKU5*) loss of function redundantly and drastically inhibited seed production and integument/seed coat development [60].

In this study, under IAA treatment, the RNA accumulation patterns of *TaFBXL* were downregulated, whereas those of *TaGPI-AP* were upregulated (Figure 6B). It is assumed that increased *TaGPI-AP* mRNA levels result in increased translation to form TaGPI-AP, which could interact with extracellular auxin by binding to ABP1 for exogenous auxin response. The decreased RNA accumulation of *TaFBXL* under exogenous IAA treatment results presumably in lower TaFBXL protein levels, thereby possibly leading to increased TaGPI-AP protein levels. TaGPI-AP is not degraded by the 26S proteasome in the presence of MG132, which effectively blocks the proteolytic activity of the 26S proteasome complex (Figure 6A). In addition, the level of TaGPI-AP was increased following MG132 treatment, which was a result of the inhibition of the 26S proteasome system. Therefore, it is assumed that the different levels of TaGPI-AP between MG132- and IAA-treated samples were a result of the difference in the amount of degradation via the 26S proteasome. Consistent with this hypothesis, more accumulation of TaGPI-AP was detected in both MG132- and IAA-treated samples than in MG132- or IAA-treated samples (Figure 6C). The RNA accumulation of *TaGPI-AP* is upregulated by exogenous auxin application, and degradation of TaGPI-AP could possibly be reduced by decreasing the abundance of TaFBXL through the 26S proteasome system. This could lead to the accumulation of TaGPI-AP in the cell, thus enabling TaGPI-AP and the auxin-related extracellular receptor ABP1 to recognize exogenous auxin. Altogether, our study suggests that TaGPI-AP is degraded by the ubiquitin–proteasome system in plant cells, and TaFBXL acts as a potential regulator of TaGPI-AP in the presence of exogenous auxin to facilitate the signal transduction of the ABP-based cell surface receptor complex.

## Figures and Tables

**Table 1:** List of TaFBXL-interacting protein candidates identified by yeast two-hybrid assays and BLASTX results searched from NCBI NR (nonredundant) database.

Clone No.	Putative Identification	Organism	*e*-Value
25, 28	WESR2 (wheat early salt-stress responding gene 2)	*Triticum aestivum*	0.00
34	Signal recognition particle 9-kDa protein	*Aegilops tauschii*	0.00
38	Chlorophyll a-b binding protein of LHCII type 1	*Aegilops tauschii*	0.00
42	40S ribosomal protein S3a	*Aegilops tauschii*	0.00
45	Glycosylphosphatidylinositol (GPI)-anchored proteins, At3g06035-like	*Aegilops tauschii*	0.00

## Supplementary Materials

The following are availble online at https://www.mdpi.com/article/10.3390/plants10081606/s1, Table S1. The information of primers used in this study. Table S2. The CDS and peptide sequence of the *TaFBXL* gene. Table S3. High-confidence domains and repeats in the TaFBXL protein.
