# Supplementary material for: Regulation of Glycosylphosphatidylinositol-Anchored Protein (GPI-AP) Expression by F-Box/LRR-Repeat (FBXL) Protein in Wheat (Triticum aestivum L.)
Source: Plants (Basel). 2021 Aug 5;10(8):1606. doi: 10.3390/plants10081606 (PMC8397982; doi:10.3390/plants10081606)
Supplement: Supplementary file 1 [file plants-10-01606-s001.zip › plants-1304391-supplementary.pdf]

Supplementary Table S1. The information of primers used in this study.

Purpose	Gene	Forward primer	Reverse primer
Gateway cloning	*TaFBXL*	ATGGTTAGTGCGAGGTCAGTGG	TCAGTACGCATGGCTGGTGGC
	TaGPI-AP	ATGGATTCCAGGGCCGCCCTCT	CTTCATCAGCAAAACTACGAA
	TaABP1	ATGGCGAGTGGACACACAACAGCTG	TTACAGTTCATCTTTAGGTGCGTCT
qRT-PCR	*TaFBXL*	TCAGCAACCTCATGGTGT	AAACACCGGATGCGACAA
	TaGPI-AP	CGACAACCAGCTCCTCAA	TTCTTTGCTAGCTGCTCG
	Actin (AB181991)	GCCACACTGTTCCAATCTA	TGATGGAATTGTATGTCGC

Supplementary Table S2. The CDS and peptide sequence of the TaFBXL gene.

TaFBXL_CDS sequence
ATGGTTAGTGCGAGGTCAGTGGACAGGCAGTTGGATACATGCTTCAGCAACCTCATGGTGTCTAGCGGC<br>GGTGGAAGGGGGCAGGCTGAAACTGGTGGCGCTATGCCTATGTTGTCAGGATGGAAGGACCTGCCAATG<br>GAGCTGCTTATGCGGATCATATCAGTTGCTGGAGATGATCGAATTGTCGTTGTCGCATCCGGTGTTTGC<br>ACTGGCTGGCGCGACGCGTTAGGACGGGGGTTACTAATCTTTCCCTTTCATGGTGCCAGCAGAACATG<br>AATAACTTAACGATATCATTTGCTCACAAGTTCACAAAGCTTCAGGTTCTCACTCTTCGCCAAATCAAA<br>CCTCAGCTCGAAGACAGTGCAGTAGAGGCTGTTGCCAACTACTGTTATGATCTACGTGAGTTAGACCTC<br>AGCCGAAGTTTTCGGCTTAGTGACCGATCGTTGTATGCATTGGCCAATGGATGTCCTCGGCTTACAAAA<br>CTGAACATCAGTGGGTGTTCCAGTTTCAGTGACAGTGCCTTAATCTACCTTAGTTGCCACTGTAAAAAC<br>CTGAAGAGCTTGAATCTTTGTGGATGTGGAAAGGCAGCCACTGATGAGTCCTTGCAGGCCATAGCCCAA<br>AACTGTGGGCACCTGCAATCTTTAAACCTAGGTTGGTGTGACAATGTCACAGATGAGGGGGTTACCAGC<br>TTGGCATCAGGCTGCCCTGATCTCAGGGCCCTGGATTTGTGTGGCTGTGTTCTTATAACAGACGAAAGT<br>GTTATTGCTCTAGCAAGCGGGTGCCCGTACCTGCGATCTTTGGGCCTGTACTACTGCCAGAACATCACC<br>GACCGTGCCATGTACTCCCTCGCAAACAGCTGCGTGAAGAGCAAACGAGGGAGGTGGGGCACCCCGCGG<br>AGCAGCAGCAGCAACTCGAAGGACGTCGACGGGCTGGCTAATCTGAACATCAGCCAGTGCACGGCCCTG<br>ACGCCTCCCGCGGTTCAGGCGGTGTGCGACTCCTTCCCGTCGCTCCACACCTGCCCGGAGAGGCACTCC<br>CTCATCATCAGCGGTTGCCTCAGCCTGACTAACGTCCACTGTGCCTGCGGCCTCCAGCGCCACCGCGCC<br>GGAAGCGCCCTGCAGGCCACCAGCCATGCGTACTGA
**TaFBXL_amino acid sequence**
MVSARSVDRQLDTCFSNLMVSSGGGRGQAETGGAMPMLSGWKDLPMELLMRIISVAGDDRIVVVASGVC<br>TGWRDALGRGVTNLSLSWCQQNMNNLTISFAHKFTKLQVLTLRQIKPQLEDSAVEAVANYCYDLRELDL<br>SRSFRLSDRSLYALANGCPRLTKLNISGCSSFSDSALIYLSCHCKNLKSLNLCGCGKAATDESLQAIAQ<br>NCGHLQSLNLGWCDNVTDEGVTSLASGCPDLRALDLCGCVLITDESVIALASGCPYLRSLGLYYCQNIT<br>DRAMYSLANSCVKSKRGRWGTPRSSSSNSKDVDGLANLNISQCTALTPPAVQAVCDSFPSLHTCPERHS<br>LIISGCLSLTNVHCACGLQRHRAGSALQATSHAY*

Supplementary Table S3. High-confidence domains and repeats in the TaFBXL protein.

Domain name	Domain start (bp)	Domain end (bp)	e-value	SMART accession no.	Definition
F-box	44	84	2.0e-9	SM000256	A receptor for ubiquitination targets

LRR	103	129	2.0e-8	SM000370	Leucine-rich repeats
LRR	130	155	4.54e-4		
LRR	156	181	1.47e-3		
LRR	182	208	4.24e-4		
LRR_CC	209	234	2.07e-7	SM000367	Leucine-rich repeat, cysteine-containing subfamily
LRR_CC	235	260	5.17e-8		
LRR_CC	261	286	2.77e-8		
LRR	308	333	3.0e-8	SM000370	Leucine-rich repeat
